# Supplementary material for: Host- and Age-Dependent Transcriptional Changes in Mycobacterium tuberculosis Cell Envelope Biosynthesis Genes after Exposure to Human Alveolar Lining Fluid
Source: Int J Mol Sci. 2022 Jan 17;23(2):983. doi: 10.3390/ijms23020983 (PMC8780516; doi:10.3390/ijms23020983)
Supplement: Supplementary file 1 [file ijms-23-00983-s001.zip › Supplemental_material_legends-FINAL.pdf]

SUPPLEMENTAL MATERIAL

FIGURES

Supplemental Fig 1. Comparison of gene expression results between targeted qPCR (qPCR) and multiplex qPCR (Biomark) for selected cell envelope genes in ALF-exposed *M.tb*. *M.tb* $H_{37}R_v$ was exposed to individual healthy human ALFs (n=6 for qPCR and n=2 for Biomark). Relative expression was calculated with the $2^{-\Delta\Delta CT}$ method (ALF-exposed *M.tb vs.* heat-inactivated ALF-exposed *M.tb*) using *rpoB* as the reference gene. Values are plotted as the means ± SEM (Prism v9). Statistical significance between the two methods was calculated for each of the genes with a two-way ANOVA using the Sidak's correction for multiple comparisons test with a 95% confidence interval, ns: non-significant.

TABLES

Supplemental Table 1. **List of primers used for multiplex qPCR assay using the Biomark HD instrument.** The table includes gene name, locus tag, product, function, pathway and functional category obtained from Mycobrowser (https://mycobrowser.epfl.ch) and KEGG (https://www.kegg.jp), sequences for forward and reverse primers, primer melting temperature (T_m), 3' complementariety, and product size.

Supplemental Table 2. Relative expression of *M.tb* cell envelope biogenesis genes. Gene expression was obtained after multiplex qPCR assay with the Biomark 96.96 IFC Dynamic Array IFC for Gene Expression (Fluidigm). Fold change values were calculated with the $2^{-\Delta\Delta CT}$ method (ALF-exposed *M.tb vs*. corresponding heat-inactivated ALF-exposed *M.tb*) using *rpoB* as the reference gene. Statistical significance of A-ALF-exposed *M.tb vs.* E-ALF-exposed *M.tb* was calculated with a two-way ANOVA for multiple comparisons with an uncorrected Fisher's LSD test. * p-value < 0.05; ** p-value < 0.005; ** p-value < 0.0005; **** p-value < 0.00005.
