# Supplementary figures and images for: Host- and Age-Dependent Transcriptional Changes in Mycobacterium tuberculosis Cell Envelope Biosynthesis Genes after Exposure to Human Alveolar Lining Fluid
Source: Int J Mol Sci. 2022 Jan 17;23(2):983. doi: 10.3390/ijms23020983 (PMC8780516; doi:10.3390/ijms23020983)

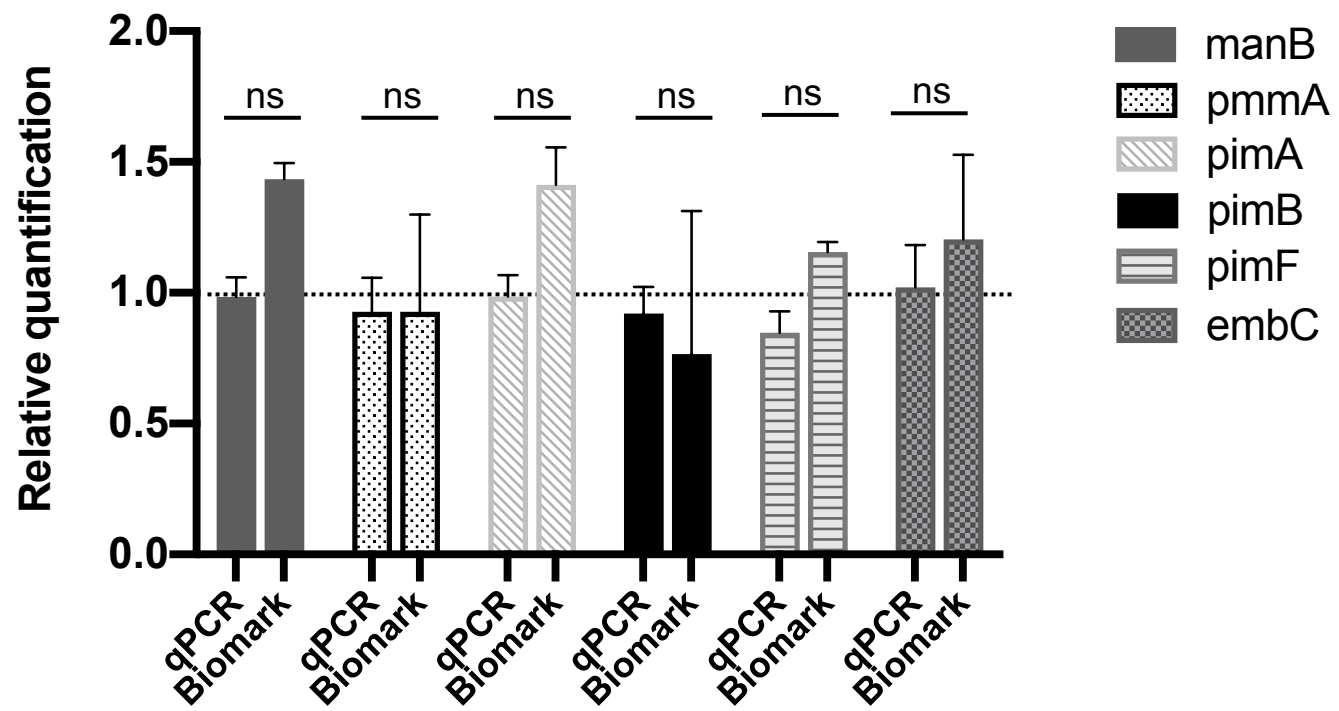

Supplement: Supplementary file 1 [file ijms-23-00983-s001.zip › Supplemental_Figure S1.pdf]
